# Molecular characterization of Coxsackievirus A2 isolated from hand, foot and mouth disease in eastern China

**DOI:** 10.1099/mgen.0.001481

**Published:** 2025-09-11

**Authors:** Liu Chen, Guifang Zhao, Huabin Wang, Zhenpeng He, Ning Chen, Wei Guo

**Affiliations:** 1 Department of Clinical Laboratory, Affiliated Jinhua Hospital, Zhejiang University School of Medicine, Jinhua, Zhejiang 321000, PR China; 2 Jinhua Institute of Zhejiang University, Jinhua, Zhejiang 321299, PR China

**Keywords:** Coxsackievirus A2, genetic characterization, genetic recombination, phylogenetic analysis

## Abstract

Coxsackievirus A2 (CVA2) belongs to the enterovirus group A. In this study, we determined the whole-genome sequences of two strains of CVA2 virus isolated from the faeces of patients with hand, foot and mouth disease in Jinhua, Zhejiang Province, China, in 2024. Their nucleotide and amino acid sequences showed 79.12%–79.50% and 94.47%–94.93% identity with the prototypical Fleetwood strain, respectively, and 97.94% and 98.17% mutual identity, respectively. Recombination analysis showed that the P2 region of the two CVA2 strains recombined with Coxsackievirus A4 (CVA4/JN19838/CHN/2019). This study obtained the whole-genome sequences of two CVA2 strains, enriching the molecular characterization of CVA2 in China.

**Impact Statement**

Clinical data in recent years have shown that the rate of Coxsackievirus A2 (CVA2) infection and mortality has been increasing, making research on this virus particularly important. In addition, clinical studies have shown that some CVA2 patients develop serious neurological complications after infection, such as encephalomyelitis, acute flaccid paralysis and cardiopulmonary dysfunction. However, relatively little is known about CVA2, and there is no effective antiviral treatment. We report the whole-genome sequences of two strains of CVA2 virus isolated from the faeces of patients with hand, foot and mouth disease in Zhejiang Province and compared the genetic characterization data of these strains with the sequences of other enterovirus group A serotypes. The results showed that the CVA2 Chinese epidemic strain was located in genotype D and had a recombination event in the P2 region of the nonstructural protein genome with the Coxsackievirus A4 strain, which had a high degree of genetic diversity. In addition, the results of selection pressure showed that the two CVA2 strains exhibited two positive selection loci, which may increase their virulence and pathogenicity. In this study, we obtained the whole-genome sequences of the two CVA2 strains, which enriched the molecular characterization of CVA2 in China, suggesting that CVA2 has become an important pathogen jeopardizing the lives of the Chinese people and providing information for future vaccine development or drug research.

## Data Summary

The dataset generated and/or analyzed in this study has been submitted to the National Center for Biotechnology Information repository under accession numbers PQ558666 and PQ558667 and is available in the supplementary text file (Supplementary Material 1).

## Introduction

Enteroviruses (EVs) belong to the genus *Enterovirus* in the family *Picornaviridae*, which consists of 15 species, divided into 327 serotypes (www.picornaviridae.com and https://ictv.global/report/chapter/picornaviridae/picornaviridae/enterovirus). Coxsackievirus A2 (CVA2) belongs to the EV group A (EV-A). CVA2 consists of an unenveloped icosahedral capsid encapsidating a single-stranded positive RNA with a genome length of ~7,400 nucleotides. The genome structure is an ORF with a UTR at both ends, and its encoded precursor proteins include three parts: P1, P2 and P3 [1]. P1 includes the structural proteins of coat protein, VP1, VP2, VP3 and VP4, while P2 and P3 include seven non-structural proteins (2A, 2B, 2C, 3A, 3B, 3C and 3D) [2]. The VP1 sequence contains serotype-specific information that can be used for viral characterization, and the complete or partial VP1 sequences have been widely used in the study of EVs [3,4].

Molecular epidemiological studies have shown that EV-A viruses are co-infecting and that EVs have extensive recombination capacity, where parts of the genome of different types may be recombined, thus enhancing the genetic diversity and adaptability of the viruses [5,6]. Other studies have shown that other non-EV 71 (EV-A71) and non-Coxsackievirus A16 (CVA16) viruses, such as CVA2, occupy a higher proportion of patients with hand, foot and mouth disease (HFMD) [7,9]. These findings suggest that viruses such as CVA2 may play an important role in the evolution of HFMD-associated EVs.

Recent studies have shown that CVA2 is one of the aetiologic agents of recent sporadic cases and outbreaks of HFMD in the Asia-Pacific region, which have been reported in China, Thailand, Japan and South Korea [10,14]. The continued spread and evolution of CVA2 has resulted in its genetic diversity, which makes the burden of disease attributable to CVA2 likely to be much higher than existing estimates. Clinical data in recent years have shown that some CVA2 patients develop serious neurological complications after infection, such as encephalomyelitis, acute flaccid paralysis and cardiopulmonary dysfunction [15,18]. In addition, CVA2 is strongly associated with the development of herpangina [19,20].

Relatively little is known about CVA2, and there is no effective antiviral treatment. With the outbreak and prevalence of CVA2, it is important to explore this virus. In this study, two CVA2 strains were found in Zhejiang Province, China, in patients with HFMD. The whole-genome sequencing and analysis of these two CVA2 strains will help us to further understand the molecular and evolutionary characteristics of CVA2.

## Methods

### Ethics statement

The study was conducted in accordance with the principles of the Declaration of Helsinki and was approved by the Ethics Committee of Jinhua Hospital, School of Medicine, Zhejiang University, in compliance with the ethical regulations of the institution under review approval number 2024-Ethics-191. The Ethics Committee of Jinhua Hospital Affiliated to Zhejiang University School of Medicine waived the requirement for informed consent because the study was retrospective in nature.

### Viral isolation

Stool samples were collected from 18 patients with clinically diagnosed acute-phase HFMD in Jinhua, Zhejiang Province, China, between April and May 2024. The virus was passaged and isolated from the Vero cell line of the original clinical faecal sample [21]. Once the cells had formed a dense monolayer, the monolayer was rinsed three times with PBS. Then, filtered and sterilized samples were inoculated onto the cell surface. After 2 h of adsorption, the appropriate amounts of maintenance solution and mycoplasma remover were added. The cells were then transferred to an incubator at 37 °C and 5% CO₂ for seven consecutive days. Samples exhibiting a cytopathic effect (CPE) were considered positive and stored at −80 °C. After three freeze-thaw cycles, the next round of passaging was performed. This blind passaging was performed for three consecutive generations. After three passages, the cultures that were positive for CPE were stored at −80 °C for future use. These cells were provided by Jinhua College of Zhejiang University.

### Viral identification and sequencing

The culture supernatants were collected and subjected to three freeze-thaw cycles in order to ensure complete release of the virus from the cells. Viral RNA was extracted from the 140 µl culture supernatants using the QIAamp Viral RNA Mini Kit (QIAGEN, California, USA) as per the instructions. The PrimeScript^™^ One Step RT-PCR Kit Ver.2 (TaKaRa, Dalian, China), with the following primer pairs AOS (CCNTGGATHAGYAACCANCAYT) and AOAS (GGRTAANCCRTCRTARAACCAYTG), was used to perform reverse transcription PCR (RT-PCR) [22] to obtain the partial VP1 region sequences. PCR-positive products were sequenced using an ABI 3130 Genetic Analyser (Applied Biosystems, Inc., USA) at Hangzhou Tsingke Biotechnology Co. (Hangzhou, Zhejiang Province, China) for sequencing. The obtained sequences were compared using blast (http://www.ncbi.nlm.nih.gov/BLAST/) for serotype determination by comparing nucleotide sequences with known ones in the GenBank database. PCR amplification was performed using the PrimeScript^™^ One Step RT-PCR Kit Ver.2 (TaKaRa, Dalian, China). At the 5′ terminus, two primer pairs (EV-1F/AOAS and A2-1F/A2-1R) were employed for long-range PCR amplification, generating 3,000 and 1,500 nt products, respectively, with subsequent bidirectional sequencing yielding 1,001/991 nt and 976/892 nt fragments. At the 3′ terminus, four primer pairs (A2-2F/EV8R-2 A2-4F/A2-3R and A2-5F/A2-2R) amplified products ranging from 1,000 to 4,600 nt, with unidirectional or bidirectional sequencing producing high-quality reads between 868 and 1,040 nt in length. The primers used for whole-genome sequencing were designed through a ‘primer walking’ strategy [23]. PCR amplification was performed under the following conditions: 55 °C for 30 min, 94 °C for 2 min, 35 cycles of 94 °C for 30 s, 52 °C for 30 s and 72 °C for X min, with 72 °C set for 7 min after the cycles. In each cycle, the extension time at 72 °C was determined by the size of the PCR product and was set at 1,000 nt/min. All primers utilized in this study are listed in Table 1. PCR products were sequenced at least twice in both directions using an ABI 3130 Genetic Analyser (Applied Biosystems, USA).

**Table 1.** Primers used for complete genome amplification and sequencing

Primer	Sequence (5′→3′)	Nucleotide position	Orientation
EV-1F	TTAAAACAGCCTGTGGGTTG	1–20	Forward
A2-1F	ACGAACATCAACTACTACAA	829–848	Forward
A2-1R	TCCAAATGCTAAGATGTA	2367–2350	Reverse
A2-2F	CACAAATGCCACAGGGTTTA	2737–2756	Forward
AOAS	GGRTAANCCRTCRTARAACCAYTG	3109–3086	Reverse
A2-3F	AGGTCACTCCGAACCAGG	3628–3645	Forward
A2-4F	CCGTATTGAACCTGTATGTC	4423–4442	Forward
A2-5F	TGTAGCCCTCTGGTGTGC	4911–4928	Forward
A2-2R	ACATTGACCTGCCTTAGTGG	5822–5803	Reverse
A2-3R	CATTCAGGCTACTGGCTT	6476–6459	Reverse
EV8R-2	GCTATTCTGGTTATAACAAATTTACC	7365–7340	Reverse

### Selection pressure analysis of the VP1 gene

VP1 sequences were accepted for selective analysis with the use of the Datamonkey online application (http://www.datamonkey.org). Four methods – mixed-effects evolutionary modelling (MEME), fixed-effects likelihood (FEL), fast unbiased Bayesian approximation (FUBAR) and single-likelihood ancestor counting (SLAC) – were employed for calculation [24]. A significance level of 0.05 was set at 0.05 for MEME, FEL and SLAC, while a posterior probability threshold of 0.9 was used for FUBAR. Results were considered credible if they were positive by at least three of the four methods.

### Sequence analysis and recombination analysis

Nucleotide and amino acid sequence analyses were performed using Geneious 10.2.2 software. Phylogenetic analyses were conducted using mega 7.0 with the Kimura two-parameter model and neighbour-joining method, employing 1,000 bootstrap replicates [25]. Recombinant sequences were identified by utilizing the default mode of the Recombination Detection Program (RDP) package Beta 4.101. The recombination analysis used seven algorithms from the program, including RDP, GENECONV, BootScan, Maxchi, Chimaera, SiScan and 3Seq [26]. Significance of recombination events obtained through three or more algorithms at *P*<0.01 was considered reliable. Strain ZJ26/ZJ/CHN/2024 was compared to other sequences listed in GenBank to determine the sequences with the highest homology to ZJ26/ZJ/CHN/2024 and the non-CVA2 sequences with the highest similarity in each genomic segment.

## Results

### Primary characterization and whole VP1 sequence analysis

Virus isolation was performed using Vero cells, and 4 positive strains were obtained from 18 HFMD patient stool samples collected between April and May 2024 for subsequent experiments. Two of the strains were CVA2 strains, designated ZJ23/ZJ/CHN/2024 (ZJ23, PQ558666) and ZJ26/ZJ/CHN/2024 (ZJ26, PQ558667). The other two strains were Echovirus 18 and Coxsackievirus A10. ZJ23 was isolated from a 3-year-old female child who had an acute onset of symptoms on 28 April 2024, with a high fever (maximal temperature of 39.6 °C), during which cyanosis of the lips was observed, and an examination revealed pharyngeal inflammation with multiple blisters in the oropharynx. The ZJ26 strain was isolated from a 9-month-old male child who presented on 4 May 2024, with an acute high fever (maximum temperature 39.8 °C), accompanied by cold extremities and oropharyngeal blisters on examination. Both children were admitted to the hospital on the basis of clinical manifestations consistent with herpes pharyngitis. Follow-up diagnostic evaluation confirmed the aetiology to be EV infection.

In this study, 154 complete VP1 sequences from GenBank were used for nucleotide and amino acid analysis. The VP1 sequences of the two Zhejiang CVA2 isolates (885 nt each) shared the highest nucleotide identity (97.06%–97.74%) and amino acid identity (98.31%–100.00%) with the CVA2 strain CVA2/Suzhou-p2022054/CHN/2022 (OQ957169), isolated from an HFMD patient in China. These two isolates shared 80.45%–81.36% nucleotide identity and 94.58%–96.27% amino acid identity with the entire VP1 sequence of Fleetwood (AY421760), the prototype CVA2 strain isolated in the USA in 1947 [27], and 93.11%–97.63% nucleotide identity and 97.29%–100.00% amino acid identity with other CVA2 strains. The whole VP1 nucleotide and amino acid identities of the two CVA2 isolates from this study were 98.19% and 98.31%, respectively.

**Fig. 1.**
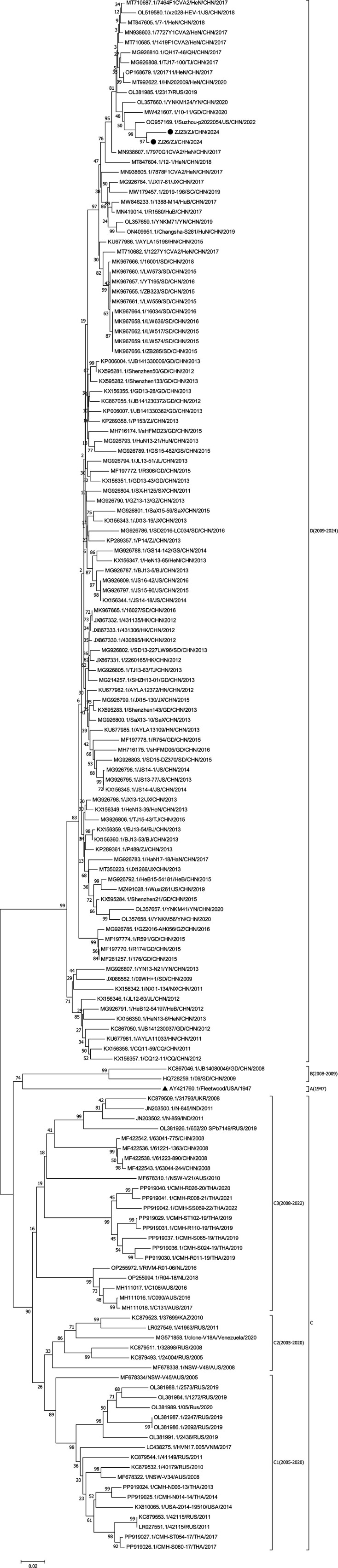
Phylogenetic tree based on the full VP1 sequence (885 bp) of the global CVA2 isolate, analysed by nucleotide sequence alignment using the neighbour-joining algorithm in the mega 7 program. Numbers on nodes indicate bootstrap support (percentage of 1,000 bootstrap repeats) for that node. Scale bars represent genetic distances. Only high bootstrap values (>75%) are shown. ▲: the prototype CVA2. ●: strains isolated in this study.

Two CVA2 isolates collected in this study were analysed (Fig. 1), including 154 complete VP1 sequences from GenBank. The strains were classified into four genotypes (A–D) based on the approximate average 25% cut-off difference value used to genotype EV and the approximate average 15% cut-off difference value used for genotyping subtypes [28,29], with genotype C being classified into three genotypic subtypes (C1–C3). The genotype A contained only one CVA2 prototype strain, Fleetwood. The genotype B consists of two Chinese isolates, JB14080046/GD/CHN/2008 (KC867046) and 09/SD/CHN/2009 (HQ728259), isolated in Guangzhou, China, in 2008 and in Shandong, China, in 2009. The predominantly endemic strains belonged to the genotypes C and D, which comprised the majority of CVA2 strains (153 strains). The genotype C was divided into three subtypes (C1–C3), and the genotype C consisted mainly of CVA2 isolates obtained from Russia, Australia, Thailand, the Republic of Kazakhstan and India from 2005 to 2022. The genotype D consisted of 106 isolates obtained in China from 2009 to 2024 and one strain isolated in Russia in 2019. The genotype D contained the two isolates in this study.

### Selection pressure analysis of the CVA2 VP1 gene

Regarding the VP1 gene, two positive selection sites were found in the CVA2 Zhejiang strains. Two positive selection sites were found at amino acid position 102 (position 102, S→N) and amino acid position 145 (position 145, R→Q) of VP1. Site 145 showed the strongest positive selection (MEME: ω^+^=188.6, *P*=0.00; FUBAR: dN/dS=4.44, Post.Pr.=0.999). Site 102 was also significant (MEME: ω^+^=95.74, *P*=0.00; FUBAR: dN/dS=3.17, Post.Pr.=0.999). Both sites were validated by SLAC for positive selection (dN/dS>2, *P*=0.02), indicating that these two sites are driven by strong positive selection pressure (Table 2).

**Table 2.** VP1-positive selection sites for CVA2

Position aa	Method							
	SLAC		FEL		MEME		FUBAR	
	dN/dS	*P*-value	dN/dS	*P*-value	ω^+^	*P*-value	dN/dS	Post. Pr.
102	2.17	0.02	/	/	95.74	0.00	3.17	0.999
145	2.53	0.02	/	/	188.6	0.00	4.44	0.999

### Whole-genome sequence analysis

The complete genome sequences of two isolates (designated ZJ23 and ZJ26), obtained in Zhejiang Province in 2024, were determined. The genome sequences span 7,364 to 7,365 nucleotides, including an ORF of 6,570 nucleotides that encodes a polyprotein of 2,190 amino acids. The ORF is flanked by a 5′-UTR of 745 nucleotides and a 3′-UTR of 49–50 nucleotides. The whole-genome nucleotide and amino acid identities between the two isolates are 97.94% and 98.17%, respectively. The base composition is as follows: A, 27.17%–27.23%; G, 23.95%–24.05%; C, 24.18%–24.31%; and T, 24.51%–24.60%. Given the high pairwise identity of the whole-genome nucleotide sequences and deduced amino acid sequences (> 97.94%), isolate ZJ26 was selected as the representative strain for further analysis.

Pairwise comparisons of the nucleotide and amino acid sequences of isolate ZJ26 with the prototype Fleetwood strain and other CVA2 strains are presented in Table 3. The whole-genome nucleotide identities of isolate ZJ26 with the prototype Fleetwood strain and other CVA2 strains are 79.50% and 79.50%–96.65%, respectively, while the derived amino acid sequence identities are 94.93% and 94.97%–98.34%, respectively.

**Table 3.** Nucleotide and amino acid sequence identity between ZJ26/ZJ/CHN/2024 and the prototype CVA2 and other CVA2 strains in all sequenced genomic regions

Genomic region	Prototype CVA2		Other CVA2 strains	
	% nucleotide identity	% amino acid identity	% nucleotide identity	% amino acid identity
5’-UTR	87.52		88.61–97.86	
VP4	79.50	95.65	89.86–96.62	95.65–98.55
VP2	80.76	97.65	81.81–98.31	97.65–100.00
VP3	82.39	96.67	82.39–95.69	96.25–98.75
VP1	81.36	96.27	93.11–97.63	97.29–100.00
2A	75.68	93.33	88.64–96.44	93.33–96.67
2B	76.43	94.95	92.26–97.31	94.95–98.99
2C	77.04	95.14	90.68–96.66	96.66–98.18
3A	80.23	96.51	89.92–96.51	95.35–96.51
3B	72.70	90.91	96.97–98.48	90.91–100.00
3C	77.09	91.80	88.52–95.45	91.8–96.72
3D	78.07	93.07	86.58–96.75	96.10–98.48
3’-UTR	92.59		93.75–95.74	
P1	81.10	96.74	80.77–96.69	95.81–99.30
P2	76.45	94.64	90.37–96.66	96.54–98.27
P3	77.79	93.09	86.29–96.24	96.15–98.14
Genome	79.50	94.93	79.50–96.65	94.97–98.34

**Fig. 2.**
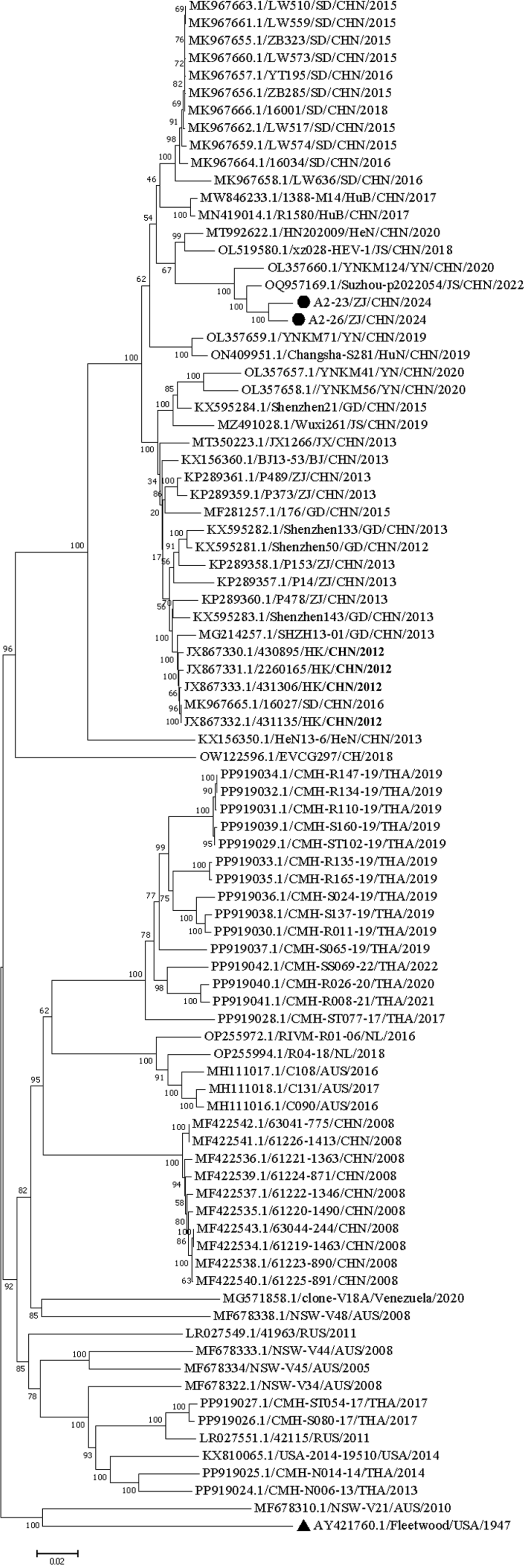
Phylogenetic relationships based on the whole-genome sequences of all CVA2 strains in the GenBank database, analysed by nucleotide sequence alignment using the neighbour-joining algorithm implemented in the mega 7 program. Numbers on nodes indicate bootstrap support (percentage of 1,000 bootstrap replicates) for that node. Scale bars represent genetic distances. Only high bootstrap values (>75%) are shown. ▲: the prototype CVA2. ●: strains isolated in this study.

Two CVA2 isolates collected in this study were analysed (Fig. 2), including 86 complete CVA2 sequences from GenBank. The results of whole-genome sequence analysis showed that the two CVA2 isolates we obtained had the highest identity to the CVA2 strain CVA2/Suzhou-p2022054/CHN/2022 (OQ957169), which was consistent with the results of VP1 analysis.

### Recombination analysis

The sequences of CVA2 and non-CVA2 strains with the highest nucleotide identities to strain ZJ26 were screened online using blast. Table 4 presents a compilation of the most similar strains observed in each segment. The 5′-UTR, VP4, 2A, 2B and 3D regions of strain ZJ26 exhibited the highest identity (96.44%–97.86%) with CVA2 strain CVA2/20HA5/CHN/2020 (PQ130039). The 2C, 3A and 3B regions showed the highest identity (96.51%–98.48%) with CVA2 strain CVA2/21HA1/CHN/2021 (PQ130040). The VP2, VP3, VP1, 3C and 3′-UTR regions demonstrated the highest identity with CVA2/7217Y1CVA2/CHN/2017 (MN938684, 98.31%), CVA2/xz028-HEV-1/CHN/2018 (OL519580, 96.69%), CVA2/7217G1CVA2/CHN/2017 (MN938600, 97.63%), CVA2/YNKM124/CHN/2020 (OL357660, 95.45%) and CVA6/Changchun046/CHN/2013 (KT779410, 97.87%), respectively.

**Table 4.** Highest similarity of nucleotide sequences of EVs in all sequenced genomic regions of the ZJ26/ZJ/CHN/2024 strain, determined with blast online

Genomic region	ZJ26/ZJ/CHN/2024		
	Strain	% nucleotide identity	Accession number
5′-UTR	CVA2/20HA5/CHN/2020 CVA4/CO19/AUS/2019	97.86 92.46	PQ130039 MH111020
VP4	CVA2/20HA5/CHN/2020 CVA6/312220005/FRA/2012	96.62 79.26	PQ130039 MT814448
VP2	CVA2/7217Y1CVA2/CHN/2017 EVB/G22-023/FRA/2016	98.31 81.28	MN938684 ON807329
VP3	CVA2/xz028-HEV-1/CHN/2018 CVA16/Shenzhen189/CHN/2017	95.69 81.25	OL519580 MH010205
VP1	CVA2/7217G1CVA2/CHN/2017	97.63	MN938600
2A	CVA2/20HA5/CHN/2020 CVA4/SDLC-16114/CHN/2016	96.44 89.71	PQ130039 MH086049
2B	CVA2/20HA5/CHN/2020 CVA4/herpangina-8/CHN/2018	97.31 94.95	PQ130039 MN964078
2C	CVA2/21HA1/CHN/2021 CVA4/JN19901/CHN/2019	96.66 95.04	PQ130040 ON730866
3A	CVA2/21HA1/CHN/2021 CVA4/JN19814/CHN/2019	96.51 95.74	PQ130040 ON730869
3B	CVA2/21HA1/CHN/2021 CVA4/JN20147/CHN/2020	98.48 92.42	PQ130040 ON730863
3C	CVA2/YNKM124/CHN/2020 CVA14/PZ05Y/CHN/2012	95.45 92.53	OL357660 KP036482
3D	CVA2/20HA5/CHN/2020 CVA8/18-38/CHN/2018	96.75 89.55	PQ130039 MT648786
3′-UTR	CVA6/Changchun046/CHN/2013	97.87	KT779410
P1	CVA2/7217G1CVA2/CHN/2017	97.69	MN938600
P2	CVA2/20HA5/CHN/2020 CVA4/JN19838/CHN/2019	96.66 92.37	PQ130039 ON730868
P3	CVA2/20HA5/CHN/2020 CVA8/HZ040/CHN/2015	96.24 87.74	PQ130039 MT648783

**Fig. 3.**
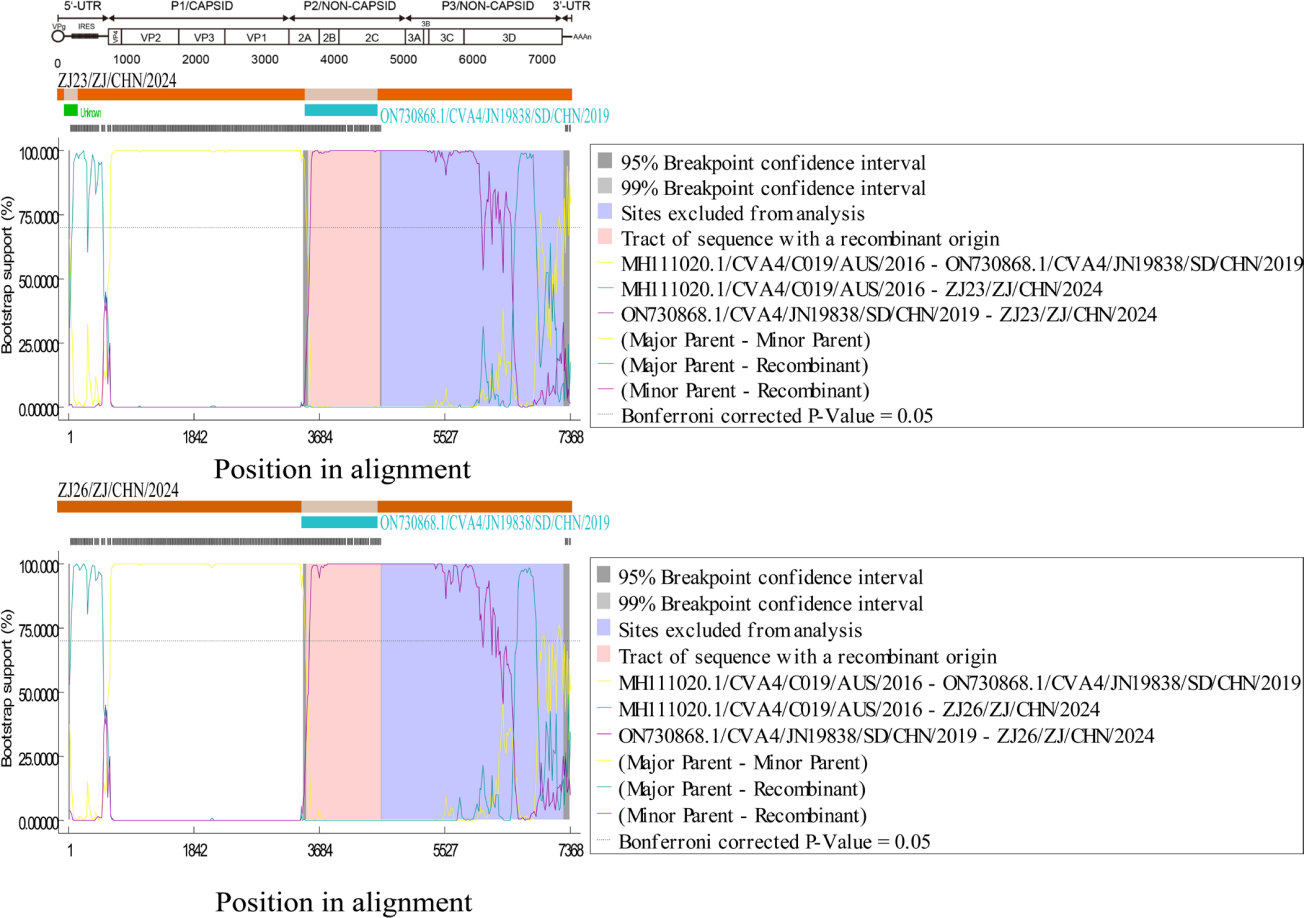
RDP4 software analysis of the strain ZJ26/ZJ/CHN/2024 with closely related strains. Seven algorithms were used for recombination analysis: RDP, GENECONV, BootScan, Maxchi, Chimaera, SiScan and 3Seq.

Strain ZJ26 showed the highest similarity (97.69%) to CVA2 strain CVA2/7217G1CVA2/CHN/2017 (MN938600) in the P1 region and to CVA2 strain CVA2/20HA5/CHN/2020 (PQ130039) in the P2 and P3 regions (>96%). In addition, RDP4 analysis showed that strain ZJ26 underwent a recombination event involving strain CVA4/JN19838/CHN/2019 (ON730868). The recombination event started in region 2A (at 3,534 nt) and ended in region 2C (at 4,584 nt), located in the P2 region of the isolate genome (Fig. 3).

## Discussion

HFMD is an infectious disease caused by EVs that primarily affects children under the age of five [30,31]. Since 2008, large-scale outbreaks of HFMD have been caused by EV-A71 and CVA16 in China [32,33]. Additionally, the prevalence of CVA6 and CVA10 has been on the rise in various regions of China since 2013 [34,36]. Consequently, in mainland China, the detection of EVs is still primarily concentrated on EV-A71, CVA6, CVA10 and CVA16 [8,37, 38]. Furthermore, the epidemiology of EVs in China is undergoing a transformation due to the introduction of the inactivated EV-A71 vaccine [8,39, 40]. In 2008, epidemics and outbreaks of CVA2 began in China, including large-scale CVA2 epidemics in Taiwan and Shandong Province [16,41]. In 2012, CVA2 infections resulted in the deaths of two young children in Hong Kong [17]. While infections with CVA2 are usually mild and self-limiting [13,14, 16, 18], in some cases, they can cause more severe outcomes, including encephalomyelitis, acute flaccid paralysis and cardiopulmonary dysfunction [8,42, 43]. Additionally, CVA2 has been documented to induce severe symptoms, including cardiac damage and pulmonary oedema, in EV animal models. Consequently, it is imperative to maintain continuous monitoring of CVA2, an EV with the potential to cause severe symptoms, and to remain vigilant for any indications of epidemics or outbreaks.

Since the identification of the prototype CVA2 strain Fleetwood (AY421760), CVA2 isolates have evolved into four genotypes, suggesting that CVA2 is genetically diverse [14,44]. Genetic analysis of the VP1 sequence of CVA2 revealed that Chinese CVA2 strains were classified into B (2008–2009), C (2008) and D (2009–2024), and the two Zhejiang CVA2 isolates in the study were located in the genotype D. This suggests that the genotype D has become the prevalent genotype in China as CVA2 continues to become prevalent in the country.

Alterations in the structural proteins of the VP1 region of EV are closely related to the virulence and pathogenicity of the virus [45,47]. Specific regions on the surface of the VP1 protein (e.g. the BC loop, EF loop and GH loop) are critical regions for the binding of the virus to the receptor of the host cell, which determines the host range and the pathogenicity of the virus [48,50]. The EF loop, situated between the folds of the β-E and the β-F folds, is a looping structure, which plays a key role in the viral immune recognition [51]. It is exposed on the surface of the virus particle and is involved in the binding process of the virus to the host cell receptor [50]. VP1-145, located in the EF loop, is in the receptor binding region at the edge of the canyon, and studies have shown that mutation of glutamine (Q) in VP1-145 was associated with increased disease severity [52,53]. The results of selection pressure showed that the two Zhejiang CVA2 isolates in this study exhibited a positive selection site for R145Q. Amino acid 145 of VP1 is thought to be the primary amino acid in clathrin responsible for binding the EV receptor P-selectin glycoprotein ligand-1 (PSGL-1) in leukocytes. When the amino acid at position 145 of VP1 is either G (glycine) or Q (glutamine), it binds readily to the PSGL-1 receptor [52,53]. This positive selection site (R145Q) tends to suggest that the current Zhejiang isolate is more virulent and pathogenic, as well as more infectious to lymphocytes. We also identified another positive site on VP1 (S102N), and studies have shown that this mutation site affects RNA envelope and RNA release [54], and further studies are needed to investigate the effects on its virulence and pathogenicity.

Recombination is an important cause of the evolution of EVs, in addition to mutations during their own replication [16,55]. We used blast online to screen for strains with the highest identity to CVA2 strain ZJ26 in different regions of the CVA2 genome, and non-CVA2 strains with >88% identity were used for recombination analysis. We found that strain ZJ26 underwent a recombination event involving strain CVA4/JN19838/CHN/2019 (ON730868), which was isolated from samples of children with an outbreak of influenza-like illness in Shandong Province, China, in 2021 [56]. CVA4 is widely prevalent in China and has been implicated in HFMD outbreaks, disseminated cases and asymptomatic infections in the mainland [57]. Genetic recombination between CVA4 and other EVs has been reported to produce new strains that may be associated with more serious diseases [58]. The recombination is located in the coding region of the non-structural protein P2, which may have an impact on the replication of viral RNA and the assembly process of viral particles [58]. In addition, patients with ZJ26 were initially identified as CVA16 in clinical diagnosis by RT-PCR. The discovery of the ZJ26 strain and its identification as CVA2 during subsequent isolation and culture suggest that EV infections tend to be mostly mixed. Co-infection with multiple EVs increases the probability of EV recombination and may lead to more severe clinical symptoms [55,58].

In conclusion, the CVA2 strain has a high degree of genetic diversity and a recombination event with the CVA4 strain in the P2 region of the genome of the non-structural proteins. The CVA2 strain has been widely prevalent in China since 2008 and co-circulates with other EVs. Recombination has driven the evolution of CVA2, and the frequency of recombination events in CVA2 will increase under the influence of mixed infections. Mutation is another important reason for the evolution of CVA2. The two CVA2 strains in this study showed two positive selection sites, which may increase their virulence and pathogenicity. This suggests that CVA2 has become an important pathogen endangering people’s lives in China and requires systematic epidemiological surveillance.

## Supplementary material

10.1099/mgen.0.001481 Supplementary Material 1.

## Abbreviations

CPE: cytopathic effect
CVA2: Coxsackievirus A2
CVA16: Coxsackievirus A16
EV-A: enterovirus group A
EVs: enteroviruses
FEL: fixed-effects likelihood
FUBAR: fast unbiased Bayesian approximation
HFMD: hand, foot and mouth disease
MEME: mixed-effects evolutionary modelling
RDP: Recombination Detection Program
RT-PCR: reverse transcription PCR
SLAC: single-likelihood ancestor counting
